# G3, GENETICS, and the GSA: Two Journals, One Mission

**DOI:** 10.1534/g3.111.000877

**Published:** 2011-09-01

**Authors:** Brenda J. Andrews, Mark Johnston, R. Scott Hawley, Paul W. Sternberg, Phillip Hieter, Tim Schedl

**Affiliations:** * Editor-in-Chief, G3: Genes | Genomes | Genetics; † Editor-in-Chief, GENETICS; ‡ President of the Genetics Society of America, 2010; § President of the Genetics Society of America, 2011; ** President of the Genetics Society of America, 2012, and; †† Chair, Publications Committee of the Genetics Society of America

With the June launch of its open-access journal G3: Genes | Genomes | Genetics, the Genetics Society of America (GSA) now offers two peer-edited journals. The missions of G3 and GENETICS are fundamentally the same: to provide a forum for timely communication of the latest findings in genetics, selected by editors who are the authors' peers. But the scopes of the two journals are different. Why offer two journals?

Since 1916, GENETICS has sought to publish significant advances in the field. To be considered for publication in the journal, the Editors have stipulated that manuscripts must *provide new insights into a biological process* or *demonstrate novel and creative approaches to an important biological problem* or *describe development of new resources, methods, technologies, or tools*. And the study must be *of interest to a wide range of genetics and genomics investigators*. In short, the Editors of GENETICS seek to attract and publish articles that they believe will have a high impact on the field.

However, the GSA recognizes that this leaves large gaps in its coverage of foundational genetics and genomic research. *Impact* is relative, and *interest* and *significance* are subjective terms; the potential significance of scientifically rigorous findings will never be realized if they remain hidden. New discoveries that advance a field, no matter its size, move science forward; emerging fields will be unable to develop if practitioners are unable to publish their findings. And genetic research increasingly relies on access to data sets; rapid publication of those data sets enables future insights. These are some of the reasons that led the GSA Board of Directors to launch G3 as GENETICS's sister journal.

G3 seeks to publish articles that describe well-executed and lucidly-interpreted genetic studies of all kinds. G3 is not bound by the subjective editorial criteria of importance, novelty, or broad appeal. The *only* criteria for publication in G3 are that the results or resources described in the manuscript are *scientifically sound* and (actually or potentially) *useful*. And not being concerned for potential impact or broad appeal allows the Editors to streamline the review process, leading to quick decisions and publication.

G3's mandate includes publishing foundational research–the cornerstones of future insights and the building blocks of our discipline. Genome maps (genetic, physical, and sequence) may not provide immediate biological insights, but they pave the way for future discoveries. If those discoveries are to be realized, such data *must* be made freely accessible to geneticists. Genome-wide association (GWAS) and quantitative trait loci (QTL) studies may not always illuminate the trait under study, but the data are likely to be useful; instead of important genetic data remaining unseen in a notebook, publication offers the potential for discovery, usefulness, and synergy. In short, G3 emphasizes experimental design and potential usefulness rather than immediate or subjective impact.

G3 offers the opportunity to publish the puzzling finding or to present unpublished results that may not have been submitted for review and publication due to a perceived lack of a potential high-impact finding. Examples of studies valuable to our scientific community and therefore worthy of consideration for publication in G3 are (1) genetic and genomic studies with organisms or aspects of biology where the audience/field is small or emerging; (2) QTL studies limited to a single population; (3) mutant or RNAi screens without extensive further mutant characterization; (4) reports of genetic and physical genome maps or collections of characterized genetic markers; (5) personal exome and genome sequencing case, disease, and population reports; and (6) genome-wide association studies and analysis, including gene expression, SNP, and CNV studies in disease and control cohorts. G3 also provides a unified home for reporting genome sequence and genetic/physical maps of any organism or cell of interest.

How is G3 different from GENETICS? Its criteria for publication are simpler: the study need only be judged scientifically sound and actually or potentially useful.

Why is G3 different from GENETICS? Because these findings need to be made available in the age of genomics, regardless of predicted or perceived significance or interest.

Why have we created yet another new journal? Because the Genetics Society of America and the Editors of GENETICS and G3 recognized the need to provide forums for observations of importance as well as for studies of obvious significance. We are fulfilling our responsibility to satisfy that need.

Join us in advancing our field: submit your best work for publication in the peer-edited journals of the Genetics Society of America.

